# Expression of Twist increases the risk for recurrence and for poor survival in epithelial ovarian carcinoma patients

**DOI:** 10.1038/sj.bjc.6603533

**Published:** 2007-01-09

**Authors:** S Hosono, H Kajiyama, M Terauchi, K Shibata, K Ino, A Nawa, F Kikkawa

**Affiliations:** 1 Department of Obstetrics and Gynecology, Nagoya Graduate University School of Medicine, Tsurumai-cho 65, Showa-ku, Nagoya, Japan

**Keywords:** Twist, prognosis, immunohistochemistry, epithelial ovarian carcinoma

## Abstract

Twist is a transcription factor that regulates the expression of tumour suppressors such as E-cadherin. We examined the distribution and expression of Twist in human epithelial ovarian carcinoma (EOC) to examine its clinical significance. Paraffin sections from EOC tissues (*n*=82) were immunostained with Twist antibody, and the staining intensity was evaluated. The clinicopathological factors examined were age, International Federation of Gynecology and Obstetrics staging, histological type, tumour grade, preoperative value of CA125, peritoneal cytology, volume of ascites and residual tumour after cytoreductive surgery. Overall survival (OS) and progression-free survival (PFS) were evaluated using the Kaplan–Meier method, and multivariate analysis was performed using the Cox proportional hazard analysis. Of the 82 carcinomas, 49 (59.8%) cases were negative for Twist immunoexpression, and 33 (40.2%) were positive immunoexpression. When categorized into negative *vs* positive expression, Twist was not associated with any of the clinicopathological parameters examined. However, positive Twist expression significantly predicted poorer OS and PFS when compared with negative expression (*P*<0.0001). In the multivariate analyses, positive Twist expression was the only independent prognostic factor for survival in this study (*P*<0.0001). Positive Twist expression seems to be a useful marker in patients with EOC likely to have an unfavourable clinical outcome.

Epithelial ovarian carcinoma (EOC) is a major cause of death among gynaecological malignancies (Brun *et al*, 2000). Epithelial ovarian carcinoma(EOC) has a poor prognosis primarily owing to its late symptomatology, its frequent association with multiple intraperitoneal disseminations and high rate of distant metastases. Patient age, disease stage, tumour type and histological grade have been shown to have a prognostic value in EOC (Kosary, 1994). Treatment for advanced EOC is difficult because of both the inability to completely resect the diffuse tumour involvement on the peritoneal surface and the eventual resistance of the tumour cells to chemotherapy. Most EOC-related deaths are caused not only by the primary tumour itself but also by subsequent peritoneal metastases. In the initial step of peritoneal metastasis, tumour cells are shed from the ovary and they spread into the peritoneal cavity. The tumour cells then may attach to the monolayered peritoneal mesothelial cells that cover the inner surface of the peritoneal cavity. Once EOC cells adhere to mesothelial cells, the tumour cells may gradually migrate through the layers of these cells, invade the local region and spread to distant organs (Coukos and Rubin, 1998; Sood and Buller, 1998; Lessan *et al*, 1999). In this multisteps metastasis, tumour cells proceed from a noninvasive to an invasive, malignant phenotype (Kajiyama *et al*, 2002, 2005). During this process, the morphology of the tumour cells changes from an epithelioid or cobblestone appearance to a fibroblastic form through a process referred to as epithelial–mesenchymal transition (EMT) (Boyer *et al*, 2000; Savagner, 2001; Vernon and LaBonne, 2004). Epithelial–mesenchymal transition (EMT) is an important event in the metastatic step. Decreased expression of E-cadherin is one of the most common indicators of EMT onset. E-cadherin which is required for the formation of stable adherens junctions, plays a critical role in the maintenance of the epithelial phenotype (Boyer *et al*, 2000; Thiery, 2002; Vernon and LaBonne, 2004).

Twist was first identified in *Drosophila melanogaster* and is a basic helix–loop–helix transcription factor that is crucial for mesoderm specification in gastrulating fly embryos and regulates craniofacial development in mice by inducing cell movement and tissue reorganization (Thisse *et al*, 1987; Leptin and Grunewald, 1990). Comijn *et al* (2001) reported that Twist represses transcription from E-cadherin promoter via the E boxes that are also targeted by Snail and SIP1. Consistently, disruption of E-cadherin-mediated cell adhesion appears to be a crucial event in the EMT from noninvasive to invasive tumour cells.

Yang *et al* (2004) reported that suppression of Twist expression in highly metastatic mammary carcinoma cells specifically inhibits their metastasis to the lung, and that elevated levels of Twist mRNA are correlated with the invasive breast cancer phenotype.

These facts prompted us to hypothesize that Twist acts as a tumour-progression factor for EOC, and that Twist may have clinical usefulness as a novel molecular target or as a prognostic indicator in the treatment of EOC. Based on this hypothesis, the present study examined the immunohistochemical expression of Twist in EOC tissues to determine whether Twist expression is correlated with clinicopathological factors or the prognosis of EOC patients.

## MATERIALS AND METHODS

### Patients and tissue samples

Eighty-two human EOC tissues were obtained from patients who underwent surgical treatment at Nagoya University Hospital between 1991 and 2004 after informed consent. The age of the patients ranged from 27 to 79 years, with a median age of 54 years. None of these patients had undergone neo-adjuvant chemotherapy before surgery. Surgical treatment consisted of total hysterectomy, bilateral salpingo-oophorectomy, omentectomy and pelvic and para-aortic lymphadenectomy. When residual tumour remained, systemic lymphadenectomy was omitted. The present series consisted of 82 carcinomas that were classified into the following histological types: 32 serous carcinomas, 11 mucinous carcinomas, 27 clear cell carcinomas and 12 endometrioid carcinomas. The histological cell types and histological grade (tumour differentiation) were assigned according to the criteria of the World Health Organization (WHO) classification (serous, endometrioid and mucinous type) or Universal grading system (clear cell type) (Shimizu *et al*, 1998). Clinical stage was assigned based on the International Federation of Gynecology and Obstetrics (FIGO) staging system. In this series, 30 cases were diagnosed with FIGO stage I tumours, 14 with FIGO stage II, 28 with FIGO stage III and 11 with FIGO stage IV tumours.

All tissue samples were fixed in 10% formalin, embedded in paraffin and routinely stained with haematoxylin and eosin for histological examination. The patients received postoperative chemotherapy with platinum plus cyclophosphamide and doxorubicin (before 1997), or platinum plus paclitaxel (after 1997) for high-risk early stage (stage I with grades 2–3; stage IC; any stage II) or advanced (stages III and IV) disease. Tumour recurrence/progression was defined based on clinical, radiological or histological diagnosis.

### Immunohistochemistry

Formalin-fixed, paraffin-embedded tissue sections were cut at a thickness of 4 *μ*m. For heat-induced epitope retrieval, deparaffinized sections in 0.01 M citrate buffer (Target Retrieval Solution pH 6.1, Dako, Kyoto, Japan) were heated three times at 90°C for 5 min using a microwave oven. Immunohistochemical staining was performed using the avidin–biotin immunoperoxidase technique using Histofine SAB-PO kit (Nichirei, Tokyo, Japan) according to the manufacturer's protocol. Briefly, endogenous peroxidase activity was blocked by incubation with 0.3% H_2_O_2_ in methanol for 15 min, and nonspecific immunoglobulin binding was blocked by incubation with 10% normal goat serum for 10 min. Sections were incubated at 4°C for 12 h with primary antibody (anti-rabbit-polyclonal, at 1 : 100 dilution, Santa Cruz Biotech, CA, USA). The sections were rinsed and incubated for 30 min with biotinylated secondary antibody. After washing, the sections were incubated for 30 min with horseradish peroxidase-conjugated streptavidin, and finally treated with 3-amino-9-ethylcarbazole in 0.01% H_2_O_2_ for 10 min. The slides were counterstained with Meyer's haematoxylin. As a negative control, the primary antibody was replaced with normal rabbit IgG at an appropriate dilution. The intensity of immunostaining for Twist was scored semiquantitatively on a four-tiered scale based on the percent positivity of stained cells as follows: For the evaluation of Twist expression, the staining intensity was scored as 0 (negative), 1 (weak), 2 (medium) or 3 (strong). The extent of staining was scored as 0 (0%), 1 (1–10%), 2 (10–50%), 3 (51%<) according to the percentage of positive staining area relative to the total tumour area. The sum of the intensity and extent scores was used as the final staining score (0–6) for Twist. Tumours having a final staining score of 3–4 or 5–6 were considered to have moderately or strongly positive expression, respectively. The scoring procedure was carried out twice by two independent observers (each blinded to the other's score) without any knowledge of the clinical parameters or other prognostic factors. The concordance rate was over 95% between the observers.

### Western blot analysis

Sample preparation and immunoblot analysis was performed as described previously (Kajiyama *et al*, 2003). Briefly, we prepared various samples of total protein from surgically resected EOC tissues after patients' informed consent. Twenty micrograms of total protein were electrophoresed on a 10% sodium dodecyl sulphate-polyacryl (SDS)–amide gel and transferred electrophoretically to an Immobilon membrane (Millipore, Bedford, MA, USA). After blocking in blocking solution (5% nonfat dry milk/0.1% Tween/PBS), the membranes were incubated with a recommended dilution of primary antibodies overnight. We used same anti-Twist-antibody (Santa Cruz Biotech, CA, USA) as used in immunohistochemical staining. We also used anti-*β*-actin antibody (Sigma, St Louis, MD, USA) as primary antibodies. The membrane was incubated with horseradish peroxidase-conjugated secondary antibody (Chemicon, Temecula, CA, USA). Proteins were visualized using enhanced chemiluminescence reagent (Amersham Pharmacia Biotech, Buckinghamshire, England) followed by exposure to X-ray film.

### Statistical analysis

The association between negative *vs* positive Twist expression and clinicopathological parameters was evaluated using the *χ*^2^-tests. The classification of Twist immunoexpression pattern into negative *vs* positive expression associated significantly with overall survival (OS); therefore, this subdivision was used for further analysis regarding overall and progression-free survival (PFS).

The univariate survival analysis was based on the Kaplan–Meier method. Comparison between the survival curves was analysed using the log-rank test. The OS was defined as the time between the date of surgery and the last date of follow-up or date of death owing to EOC. The PFS was defined as the time interval between the date of surgery and the date of progression/recurrence or date of last follow-up. The prognostic significance of negative Twist expression concerning other pathological variables was assessed using the multivariate Cox's proportional hazard's analysis. Stat View software ver.5.0 (SAS, Institution Inc., Cary, NC, USA) was used for all statistical analyses and a *P*-value of <0.05 was considered significant.

## RESULTS

### Immunohistochemical detection of Twist in EOC tissues

**Figure 1:**
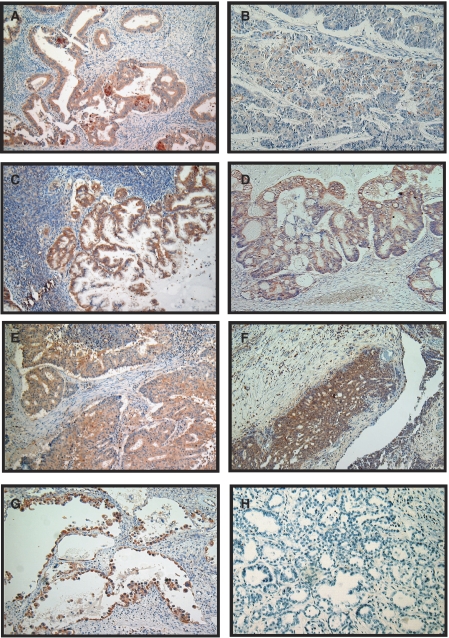
Immunoreactivity of Twist observed in EOCs. (**A**–**G**) Positive expression of Twist in EOCs. (**A**, **B**) Endometrioid cystadenocarcinoma, (**C**, **D**) mucinous cystadenocarcinoma, (**E**, **F**) serous cystadenocarcinoma, (**G**) clear cell adenocarcinoma, (**H**) negative expression of Twist (clear cell adenocarcinoma); magnification × 100.

**Figure 2:**
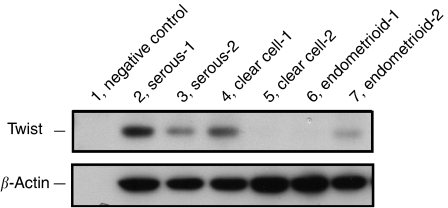
Immunoblot analysis of Twist expression in various EOC tissues. Lane 1, negative control (no protein loading); lanes 2 and 3, serous cystadenocarcinoma; lanes 4 and 5, clear cell adenocarcinoma; lanes 6 and 7; endometrioid adenocarcinoma.

As shown in Figure 1, Twist immunoreactivity was detected at various levels in EOC. There was little immunoreactivity of Twist in the tumour stroma. Of the 82 EOC specimens examined in this study, 33 (40.2%) cases were positive for Twist immunoreactivity, of which seven cases were strongly positive. Positive expression of Twist was observed in the following histological types: 14 serous types (14 out of 32; 43.8%), 13 clear cell types (13 out of 27; 48.1%), two endometrioid types (two out of 12; 16.7%) and four mucinous types (four out of 11; 36.4%). The Twist immunoreactivity categorised into negative *vs* positive (moderate and strong comined) expression was not associated with any of the clinicopathological parameters tested: age, FIGO stage, histological type, tumour grade, preoperative value of CA125, the intraoperative volume of ascites, residual tumour after primary cytoreductive surgery or peritoneal cytology (Table 1). Moreover, to show the validation of the antibody with respect to the recognition of Twist protein, we also performed an immunoblot analysis using same Twist antibody. Figure 2 shows Twist expression in various EOC tissues including several histological types.

### Correlation of Twist expression with survival of EOC patients

The median OS of the patients was 117 months (range 6–513). At the end of the follow-up period, 40 (48.8%) patients were alive, 35 (42.7%) patients were dead of the disease and seven (8.5%) patients were alive with the disease. None of the patients were lost for follow-up. During the follow-up period, the total number of cases in which death and progression/recurrence were observed was 35 (42.7%).

**Figure 3:**
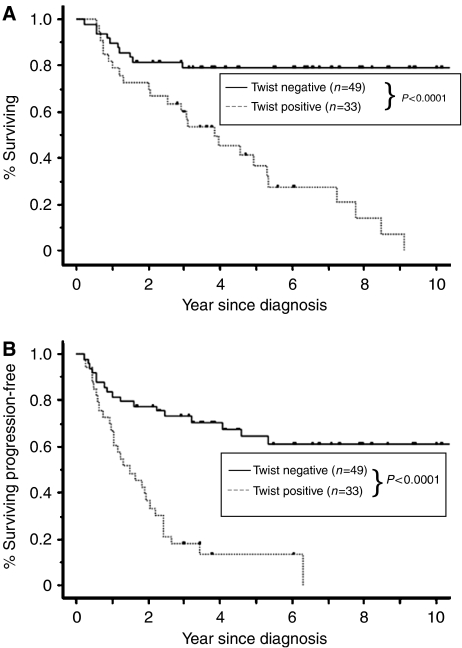
Kaplan–Meier survival curves for primary EOCs according to immunoexpression of Twist. (**A**) Overall survival (OS), (**B**) progression-free survival (PFS). Discontinuous line represents negative Twist expression (*n*=49). Continuous line represents positive Twist immunoexpression (*n*=33). Patients with Twist expression had a significantly worse carcinoma-specific survival (**A**; OS, *P*<0.0001, **B**; PFS, *P*<0.0001).

The 5-year OS rates of patients who were negative (*n*=49) and positive (*n*=33) for expression of Twist were 79.2 and 36.8%, respectively (Table 2). In univariate analyses, FIGO stage, residual tumour after primary cytoreductive surgery and positive expression of Twist were significant predictors of poor OS (Table 2). In addition, for PFS, five prognostic factors, that is, FIGO stage, histological type, residual tumour after primary cytoreductive surgery, positive peritoneal cytology and positive expression of Twist, were significant (Table 2). Figure 3 shows the OS and PFS curves with respect to Twist expression. Both OS and PFS in patients positive for expression of Twist were significantly lower than those in patients who were negative for Twist expression (OS, PFS; *P*<0.0001).

**Figure 4:**
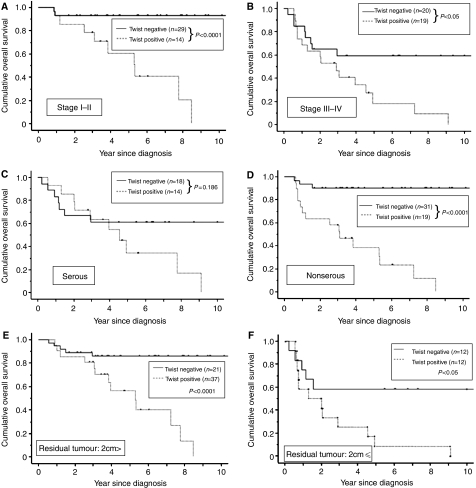
Carcinoma-specific OS rate of EOC patients with Twist expression. Kaplan–Meier survival curves for primary EOCs according to immunoexpression of Twist stratified with FIGO stage and histological type. (**A**, **B**) Kaplan–Meier survival curves stratified with FIGO stage (stages I–II (**A**; *n*=43) *vs* III–IV (**B**; n=39). (**C**, **D**) Kaplan–Meier survival curves stratified with histological type (serous type (**C**; *n*=32) *vs* nonserous type (**D**; *n*=50)). (**E**, **F**) Kaplan–Meier survival curves stratified with residual tumour after optimal debulking surgery (>2 cm (**E**; *n*=58) *vs* ⩽2 cm (**F**; *n*=24)). Discontinuous line represents negative Twist expression. Continuous line represents positive Twist immunoexpression. Stages I–II-, III–IV-, nonserous type-, >2 cm residual tumour and ⩽2 cm residual tumour with Twist expression had a significantly worse carcinoma-specific survival (*P*<0.0001, *P*<0.05, *P*<0.0001, *P*<0.0001 and *P*<0.05, respectively).

We further divided all cases into two groups according to FIGO stage (early stage; stage I+II, advanced stage; stage III+IV). There was a significant difference for OS between the two groups (stage I+II; *P*<0.0001, advanced stage group; *P*<0.05, respectively) (Figure 4A and B). Similarly, all cases were stratified according to histological subtype (serous and nonserous type) and residual tumour after primary cytoreductive surgery (>2 and ⩽2 cm). The expression of Twist was a significant prognostic factor in non-serous type although not in serous type (OS; *P*<0.0001 and *P*=0.186, respectively) (Figure 4C and D). Subsequently, we showed that there was also a significant difference for OS in the two groups with residual tumour size, less than or greater than smaller than or larger than 2 cm (>2 cm; *P*<0.0001, ⩽2 cm; *P*<0.05) (Figure 4E and F).

### Multivariate analysis of prognostic variables in EOC patients

In multivariate OS analyses, FIGO stage, residual tumour and Twist immunoreactivity were entered into the Cox proportional hazard analysis. Only the expression of Twist retained its significance as an independent prognostic factor of poor OS. The relative risk (RR) for positive Twist expression was 4.489, 95% CI, 2.113–9.537; *P*<0.0001 (Table 3). Furthermore, multivariate analysis for PFS including FIGO stage, histological type, residual tumour, positive peritoneal cytology and Twist immunoreactivity revealed that FIGO stage and positive Twist expression were independently significant prognostic factors (FIGO stage: RR, 3.861; 95% CI, 1.615–9.231; *P*<0.0001; positive immunoexpression of Twist: RR, 4.436; 95% CI, 2.275–8.652; *P*<0.0001) (Table 3).

## DISCUSSION

There have been various studies demonstrating the involvement of Twist as a crucial regulator of the metastatic process in certain malignancies; however, most of those studies were conducted in *in vitro* models or in animals (Wang *et al*, 2004; Yang *et al*, 2004; Elias *et al*, 2005; Mironchik *et al*, 2005). In the current study of 82 EOC patients with long-term follow up, high levels of Twist expression were observed in 40.2% (33 in 82) of the EOC samples, and the Twist level was not associated with any of the several clinicopathological parameters examined. High expression of Twist was associated with poor survival in both univariate and multivariate analyses of all factors that influenced survival. There have been a few other reports showing a correlation between Twist expression and clinical significance in patients with malignancies. Consistent with our result, Kyo *et al* (2006) demonstrated that Twist expression in endometrial cancers was significantly associated with the depth of myometrial invasion and shortened survival. Regarding the mechanism, one possible explanation of our current finding that Twist expression was linked with poor prognosis is that this poor prognosis may be partially owing to reduced expression of E-cadherin and increased metastatic potential of EOC. However, recent reports showed that there is a close relationship between Twist expression and the resistance to paclitaxel.

Paclitaxel is a first-line chemotherapeutic agent that is effective for the treatment of EOC. Despite the comparatively high sensitivity of EOC to paclitaxel, the prognosis of advanced or recurrent cases remains poor as most deaths result from metastasis that is refractory to conventional chemotherapy. Paclitaxel exerts its effect through stabilisation of microtubules, induction of cell cycle arrest in G2–M and activation of proapoptotic signalling (Horwitz, 1994; Wang *et al*, 2000). Maestro *et al* (1999)reported that the expression of Twist was associated with p53-induced growth arrest and that this effect was correlated with the ability of Twist to interfere with the activation of a p53-dependent reporter and to impair the induction of p53 target genes in response to DNA damage. In addition, Wang *et al* (2004) demonstrated that increased Twist was responsible for the development of acquired Paclitaxel resistance in nasopharyngeal carcinoma cells and ectopic expression of Twist conferred resistance to microtubule-disrupting agents, including paclitaxel. Although, our current cases were not always treated with paclitaxel alone, unfavourable prognostic outcome in Twist-expressing EOC patients may be owing to the role of Twist in p53-induced drug resistance as well as high metastatic potential to the peritoneum, lymphatic/vascular canal and other parenchymal organs.

Clinically, our stratification analysis showed that Twist expression is a significant risk factor even in stage I/II-EOC, as well as in stage III/IV-EOC. Furthermore, in cases with residual tumour diameter less than 2 cm, the OS of Twist-positive patients was significantly worse compared to that of Twist-negative patients (OS; *P*<0.0001). In the current cases, we observed stage-I patients with high expression of Twist who displayed distant metastasis in organs such as liver, lung and brain within 1 or 2 years after debulking surgery. Additionally, although not all of the patients in the current study received retroperitoneal lymphadnectomy, 31 cases were shown to be without pelvic and para-aortic lymph node metastasis after complete surgical staging procedures. Among these cases, all of the 20 patients whose tumours did not express Twist were alive, whereas in contrast, 63.6% (seven out of /11) of patients whose tumours expressed Twist were dead. According to these results, cases with high expression of Twist should be followed up carefully even if they are early stage, after optimal operations and/or without lymph node metastasis. Therefore, although the prognostic value of Twist needs to be confirmed in a larger number of patients, our results suggest that the immunohistochemical expression of Twist in primary EOC may be a useful marker for selecting patients with a high risk of suffering an unfavourable clinical outcome and for stratifying them for better therapeutic strategies.

Based on morphological criteria, there are four major types of primary EOC, and the chemosensitivity and biological behaviours differ among these histologic types. The stratified results of histological subtyping suggest that Twist is an important prognostic factor in nonserous adenocarcinoma (OS; P<0.0001). In addition, the survival of Twist-positive patients with serous adenocarcinoma tended to be shorter than that of Twist-negative patients, although the difference was not significant (OS; *P*=0.186). However, Twist might also become a prognostic factor even in serous adenocarcinoma if a larger numbers of cases were analysed. In general, most patients with serous adenocarcinoma already display intraperitoneal metastasis at the time of initial diagnosis because it is the character of this histological type to readily disseminate to the peritoneum at an early time. In contrast, histological subtypes such as clear cell carcinoma and mucinous adenocarcinoma tend to have a lower response rate to anticancer drugs, including paclitaxel. Although there is a definite difference among these histological types, it is possible that Twist may be a crucial factor determining the chemosensitivity or tumour behaviour. We would like to analyse Twist expression in a larger number of patients stratified according to individual histological types.

In summary, the clinical evidence obtained in this study gives support for a possible mechanism for EOC metastasis. If our findings are validated by prospective studies, Twist expression could be useful as a predictive factor for recurrence or metastasis of EOC. This could improve the current staging of EOC by defining additional criteria for the administration of systemic therapy in patients without obvious signs of advanced disease. Perhaps most important is the identification of Twist expression as a novel target for clinical therapy.

## Figures and Tables

**Table 1:** Relationship between the expression of Twist and clinicopathological parameters in EOC

		**Twist**		
	**No.**	**Negative *n***	**Positive *n***	***P*-value**
*Total*	82	49	33	
				
*Age (years)*				0.445
>55 y	43	24 (55.8%)	19 (44.2%)	
⩽55 y	39	25 (64.1%)	14 (35.9%)	
				
*FIGO stage*				0.136
I+II	43	29 (67.4%)	14 (32.6%)	
III+IV	39	20 (51.3%)	19 (48.7%)	
				
*Histological type*				0.605
Serous	32	18 (56.3%)	14 (43.7%)	
Nonserous	50	31 (62.0%)	19 (38.0%)	
				
*Grade*				0.295
G1	33	22 (66.7%)	11 (33.3%)	
G2/G3	49	27 (55.1%)	22 (44.9%)	
				
*Residual tumour (cm)*				0.247
>2 cm	58	37 (63.8%)	21 (36.2%)	
⩽2 cm	24	12 (50.0%)	12 (50.0%)	
				
*CA125* (*Uml*^−*1*^)				0.987
>35	10	6 (60.0%)	4 (40.0%)	
⩽35	72	43 (59.7%)	29 (40.3%)	
				
*Peritoneal cytology*				0.0838
Negative	29	21 (72.4%)	8 (27.6%)	
Positive	53	28 (52.8%)	25 (47.2%)	
				
*Ascitic fluid volume (ml)*				0.163
>100 ml	40	27 (67.5%)	13 (32.5%)	
⩽100 ml	42	22 (52.4%)	20 (47.6%)	

EOC= epithelial ovarian carcinoma; FIGO= International Federation of Gynecology and Obstetrics.

**Table 2:** Univariate analyses of various clinicopathological parameters in relation to survival of patients with EOC

		**Progression-free survival**		**Overall survival**	
**Variable**	**No. 82**	**5-year survival (%)**	***P*-value**	**5-year survival (%)**	***P*-value**
*Age (years)*					
>55	43	56.1	0.0711	52.4	0.3047
⩽55	39	64.2		64.2	
					
*FIGO stage*					
I–II	43	66.8	>0.0001	82.8	>0.001
III–IV	39	17.8		40.0	
					
*Histological type*					
Serous	32	21.7	0.0024	51.5	0.2161
Nonserous	50	58.9		69.6	
					
*Grade*					
G1	33	47.5	0.4362	68.4	0.2323
G2/G3	49	41.4		57.0	
					
*Residual tumour (cm)*					
>2 cm	58	51.3	0.0096	75.1	>0.001
⩽2 cm	24	25.0		33.8	
					
*CA125* (*Uml*^−*1*^)					
>35	10	78.7	0.0979	76.2	0.1926
⩽35	72	38.4		59.9	
					
*Peritoneal cytology*					
Negative	29	65.0	0.0108	75.1	0.1646
Positive	53	31.2		54.5	
					
*Ascitic fluid volume (ml)*					
>100 ml	40	50.4	0.2932	66.8	0.4405
⩽100 ml	42	37.2		54.5	
					
*Twist expression*					
Negative	49	64.1	>0.0001	79.2	>0.0001
Positive	33	13.0		36.8	

EOC= epithelial ovarian carcinoma; FIGO= International Federation of Gynecology and Obstetrics.

**Table 3:** Multivariate analyses of several clinicopathological parameters in relation to survival of patients with EOC

	**Progression-free survival**		**Overall survival**	
**Variable**	**Relative risk (95% CI)**	***P*-value**	**Relative risk (95% CI)**	***P*-value**
*FIGO stage*				
I–II	1	>0.0001	1	0.1648
III–IV	3.861 (1.615–9.231)		1.879 (0.772–4.574)	
				
*Histological type*				
Serous	1	0.8083	(—)	
Nonserous	0.930 (0.395–2.065)		(—)	
				
*Residual tumour (cm)*				
>2 cm	1	0.8406	1	0.1257
⩽2 cm	1.081 (0.507–2.305)		1.905 (0.835–4.347)	
				
*Peritoneal cytology*				
Negative	1	0.3915	(—)	
Positive	1.391 (0.654–2.960)		(—)	
				
*Twist expression*				
Negative	1	>0.0001	1	>0.0001
Positive	4.436 (2.275–8.652)		4.489 (2.113–9.537)	

EOC= epithelial ovarian carcinoma; FIGO= International Federation of Gynecology and Obstetrics.
